# Lipidomic study of cell lines reveals differences between breast cancer subtypes

**DOI:** 10.1371/journal.pone.0231289

**Published:** 2020-04-14

**Authors:** Finnur Freyr Eiriksson, Martha Kampp Nøhr, Margarida Costa, Sigridur Klara Bödvarsdottir, Helga Margret Ögmundsdottir, Margret Thorsteinsdottir

**Affiliations:** 1 Faculty of Pharmaceutical Sciences, University of Iceland, Reykjavík, Iceland; 2 Faculty of Medicine, University of Iceland, Reykjavík, Iceland; 3 ArcticMass, Reykjavík, Iceland; 4 Biomedical Center, University of Iceland, Reykjavík, Iceland; Wayne State University, UNITED STATES

## Abstract

Breast cancer (BC) is the most prevalent type of cancer in women in western countries. BC mortality has not declined despite early detection by screening, indicating the need for better informed treatment decisions. Therefore, a novel noninvasive diagnostic tool for BC would give the opportunity of subtype-specific treatment and improved prospects for the patients. Heterogeneity of BC tumor subtypes is reflected in the expression levels of enzymes in lipid metabolism. The aim of the study was to investigate whether the subtype defined by the transcriptome is reflected in the lipidome of BC cell lines. A liquid chromatography mass spectrometry (LC-MS) platform was applied to analyze the lipidome of six cell lines derived from human BC cell lines representing different BC subtypes. We identified an increased abundance of triacylglycerols (TG) ≥ C-48 with moderate or multiple unsaturation in fatty acyl chains and down-regulated ether-phosphatidylethanolamines (PE) (C-34 to C-38) in cell lines representing estrogen receptor and progesterone receptor positive tumor subtypes. In a cell line representing HER2-overexpressing tumor subtype an elevated expression of TG (≤ C-46), phosphatidylcholines (PC) and PE containing short-chained (≤ C-16) saturated or monounsaturated fatty acids were observed. Increased abundance of PC ≥ C-40 was found in cell lines of triple negative BC subtype. In addition, differences were detected in lipidomes within these previously defined subtypes. We conclude that subtypes defined by the transcriptome are indeed reflected in differences in the lipidome and, furthermore, potentially biologically relevant differences may exist within these defined subtypes.

## Introduction

Breast cancer (BC) is mainly diagnosed using routine mammography and self-examination. Recent large scale retrospective studies of Norwegian, European and North American women indicated that these routine examinations had little or no impact on BC mortality [1, 2]. The treatment and subsequent outcome for the patient is dependent on the underlying BC subtype. Therefore, there is a need for novel noninvasive tools for identification of BC subtype at an early stage to enable informed treatment decisions.

BC represents a group of diverse subtypes with genetic, clinical and molecular differences resulting in different proliferation and metastatic potential. Based on transcriptomic analysis BC is divided into five main subtypes. In clinical practice this information is used to identify subtypes by determining hormone receptor status of the estrogen receptors (ER) and the progesterone receptors (PgR) and whether human epidermal growth factor receptor 2 (HER2/*neu*) is amplified. The subtypes are: I) luminal A (ER+, PgR+, HER2-), II) luminal B (ER+, PgR+, HER2-/+), III) HER2-overexpressing (ER-, PgR-,HER2+), IV) triple negative breast cancer (TNBC; ER-, PgR-, HER2-) and, V) normal-like subtype [3–5]. Even though the BC tumors are classified according to these subtypes, several studies categorize BC cell lines differently and there seems to be a lack of consistency in classification of the BC subtypes [6]. Furthermore, it has been reported that high heterogeneity also exists within individual BC subtypes [5, 7].

Lipids are essential in many cellular functions related to carcinogenic pathways [8–10]. Glycerophospholipids (GPL) are important signaling molecules and have been shown to be involved in regulation of migration, apoptosis and neurotransmission [8, 9, 11, 12], and diacylglycerols (DG) are second messengers involved in apoptosis and mediate signal transduction in cancer cells [10, 13]. Multiple lipid metabolism enzymes have been investigated as potential targets for cancer therapy [14]. Altered expression of enzymes involved in lipid synthesis, storage, activation and degradation has been identified in breast tumors [11, 12, 15–17]. Mutations in the tumor suppressor *TP53* gene play a major role in carcinogenesis and cancer progression [13, 18] by several mechanisms, including effects on metabolism. Thus *TP53* has been shown to regulate glucose metabolism and modulate the expression of fatty acid synthase (FASN) involved in lipid synthesis [13, 18] and *TP53* mutation leads to changes in phosphatidylinositol acyl chain composition [19]. Dysfunctional *TP53* indicates poor outcomes for BC patients, irrespective of BC subtype, especially in PgR-negative tumors [13, 18]. Heterogeneity between the BC tumor subtypes is also reflected in the alterations of mRNA and/or protein expression levels of enzymes involved in lipid metabolism [16, 17, 20] and subtype-specific lipid profiles have been reported [21]. Collectively, these studies indicate that TNBC rely more on uptake and storage of exogeneous fatty acids (FA), whereas the luminal subtypes upregulate *de novo* FA synthesis and oxidation [17]. HER2 subtypes rely on *de nov*o FA synthesis as well as increased storage and oxidation of FA [16, 17, 20]. These lipid metabolism pathways are linked to other metabolic pathways by energy consumption and supply of building blocks to drive lipid synthesis. Glutamine and glucose metabolism provides acetyl-CoA which is a precursor for FA and lipid derivatives [10] demonstrating the complexity of predicting lipid metabolism. A few studies have focused on comparing lipid content of different BC cell lines to a non-malignant reference cell line [22–25]. These studies mainly found changes between BC cells and reference cells in GPL, not related to the underlying BC subtype [22–25]. Interestingly, Cifkova *et al*. described several specific lipids with a different abundance in BC cells compared to normal cells as well as between human BC tissues and surrounding normal tissues. They demonstrated that changes observed in BC tissues are mainly caused by different lipidomic profiles of tumor cells and that these changes correlated with the lipidomics composition of the individual BC cell lines [23].

Collectively, these studies indicate that the diversity in lipid metabolism on the mRNA and protein level is indeed reflected in the lipidome. Therefore, we hypothesize that the lipidome can be used to identify BC subtypes. We test this using human BC cell lines and investigate whether the subtypes defined by the transcriptome are reflected in the lipidome of BC cells and whether further subgroups can be detected within previously known BC subtypes. This knowledge would in turn provide the opportunity for non-invasive diagnostic tools (on e.g. plasma samples) and improved accurate diagnosis of BC patients for personalized subtype-specific treatment. We used a UPLC-QTOF-MS platform to analyze the lipidome of six cell lines derived from human BC carcinomas representing different BC subtypes.

## Methods and materials

### Materials

All cell lines were obtained from American type culture collection (ATCC, Manassas, VA, USA). SPLASH® Lipidomix® Mass Spec Standard was purchased from Avanti Polar Lipids (Alabaster, AL, USA). All chemicals were from Sigma Aldrich (St. Louis, MO, USA), unless otherwise stated. Culture flasks and plasticware were from Becton Dickinson (BD, Franklin Lakes, NJ, USA). Culture media, except H14 media, were from Gibco Life Technologies (by Fisher Scientific Company, Toronto, ON). Ultra-high purity water was prepared using a Milli-Q waters purification system (Millipore corp., Billerica, MA, USA).

### Cell lines

The cell lines used in this study were selected based on their similarities with different BC tumour subtypes (**Table 1**). Two BC cell lines are ER- and PgR-positive and HER2 negative (MCF7 and T-47D) and CAMA-1 is ER-positive, PgR-positive/negative and HER2-negative, these are all considered luminal subtypes [6, 26–28]. Two cell lines (MDA-MB-231 and MBA-MB-436) belong to the TNBC subtypes, in addition MDA-MB-436 has a *BRCA1* mutation [6, 29]. The HER2-overexpressing subtype is represented by SK-BR-3 cell line [6, 26–28]. The cell lines also differ in *TP53* mutation status with T-47D and MDA-MB-231 expressing the mutated protein (**Table 1**). MCF10A was derived from fibrocystic disease [26] and represents a non-cancer reference cell line. Since there is a lack of consistency in classification of the luminal BC subtypes into A and B, we collectively regard these cell lines as luminal subtype. Cultures were tested for mycoplasma every two months.

**Table 1:** Overview of breast cancer cell line characteristics.

Cell line	ATCC® No.	Type of tumour ^[^^30^^]^	Original tissue ^[^^30^^]^	Cell type ^[^^30^^]^	ER status	PgR status	HER2 status	*TP53*^[^^27^^]^	Subtype	Ref.
MCF 10A	CRL-10317^TM^	NT	Fibrocystic disease	Epithelial	-	-	-		NT	[26, 27, 31]
MCF7	HTB-22^TM^	AC	MS, PLE	Epithelial	+	+	-		Luminal	[6, 31–33]
T-47D	HTB-133^TM^	DC	MS, PLE	Epithelial	+	+	-	^M^	Luminal	[26, 27, 32, 34]
CAMA-1	HTB-21^TM^	AC	MS, PLE	WLELP	+	+/-	-		Luminal	[6, 26, 27]
MDA-MB-436	HTB-130^TM^	AC	MS, PLE	PMMCC	-	-	-		TNBC	[6, 26, 27]
MDA-MB-231	HTB-26^TM^	AC	MS, PLE	Epithelial like	-	-	-	^M^	TNBC	[16, 26, 27, 31, 33, 34]
SK-BR-3	HTB-30^TM^	AC	MS, PLE	Epithelial	-	-	+		HER2	[6, 31–33]

AC: adenocarcinoma, DC: ductal carcinoma, MS: metastatic site, PLE: pleural effusion, NT: Non-tumorigenic, PMMCC: Pleomorphic with multinucleated component cells, WLELP: weakly luminal epithelial-like phenotype. TP53 mutational status: ^M^ mutant protein.

### Cell culture

Culture media for MCF7, CAMA-1, MDA-MB-231, MDA-MB-436, and T-47D was RPMI-1640 (Gibco Life Technologies). SK-BR-3 cells were grown in McCoy’s 5 (Gibco Life Technologies) and MCF10A cell were cultured in H14 media [35]. All media were supplemented with 10% FBS. In addition, the medium used for T-47D was supplemented with 5 μL/mL insulin. During cultivation, the medium was replaced every 2–3 days. The incubator was kept at 37°C and 5% CO_2_. Cells were harvested when the culture had reached 70% confluence, i.e. towards the end of the proliferative phase. Cells were detached by trypsin/EDTA solution (0.25% w/v). Soybean trypsin inhibitor (10 mg/mL) was added to inactivate the trypsin. The cells were washed with phosphate buffered saline (PBS) followed by centrifugation for 3 min at 2000 rpm and PBS removed. The cell pellet was resuspended in 2 mL of PBS at a cell count of 1 million cells. Biological replicates of each cell line were harvested from three individual culture flasks (3 biological replicates).

### Sample preparation

SPLASH® Lipidomix® Mass Spec Standard (Avanti Polar Lipids) was used as a reference standard for verification of lipids retention time. The standard contains one deuterated lipid (7–9 deuterium for each lipid) from the subclasses phosphatidylcholines (PC), phosphatidylethanolamines (PE), phosphatidylglycerols (PG), phosphatidylserines (PS), phosphatidylinositols (PI), Phosphatidic acids (PA), lysophosphatidylcholines (LPC), lysophosphatidylethanolamines (LPE), cholesterol ester, monoacylglycerols (MG), sphingomyelins (SM), diacylglycerols (DG), triacylglycerols (TG), and cholesterol. The cell pellets were extracted using a modified Folch method [36]. Briefly, cell samples were thawed and transferred to a glass tube. Samples were centrifuged and PBS aspirated. The cells were extracted twice into cold chloroform/methanol/water (1:1:1, v/v/v). Before the extraction of the organic phase, 10 μL of the reference standard was added to each sample. Both chloroform bottom layers were combined, and the solvent was evaporated under a stream of N_2_ gas. The dried lipids were reconstituted into 10 μL chloroform/methanol (1:1, v/v) and diluted 10x with isopropanol/acetonitrile/water (2:1:1, v/v/v) for ultra-performance liquid chromatography quadrupole time of flight mass spectrometry (UPLC-QTOF-MS) analysis. A sample from each biological replicate (three) for each cell line was prepared to be analyzed in triplicate (9 analyses for each cell line). Quality controls (QC) were prepared by pooling all the samples. 11 QC samples were included within the sample sequence.

### UPLC-QTOF-MS settings

The setup of UPLC-QTOF-MS method was based on previously published method by Castro-Perez and colleagues [37]. The lipid samples were analyzed using Acquity UPLC (Waters corp., Milford, USA), coupled to a Synapt G1 mass spectrometer (Waters corp., Manchester, UK) equipped with electrospray ionization (ESI) probe in MS^E^ acquisition mode. The analytical column ACQUITY UPLC HSS T3 1.8 μm (2.1 mm x 100 mm) (Waters corp., Milford, USA) was used for separation. Mobile phase A was acetonitrile:water (40:60 v/v) and mobile phase B was isopropanol:acetonitrile (90:10 v/v), both supplemented with 10 mM ammonium acetate (pH 5.0). The flow rate was maintained at 0.4 mL/min. A linear gradient was used from 40 to 100% B during the first 10 min, followed by a column clean up at 100% B for 2 min and reconditioning at the initial conditions for 2.5 min. The total chromatographic run time was 14.5 min. The sample manager temperature was maintained at 4.0°C. The capillary voltage was set to 3.0 kV, the cone voltage to 35 V and extraction cone to 4.0 V. The scan time was 0.1 seconds in the mass range of 100–1000 Dalton. Source temperature 120°C; desolvation temperature 400°C at a flow rate of 800 L h^-1^ (N_2_) and cone gas flow rate 50 L h^-1^. The data was captured as centroid data with a resolution of 9000 (full width at half maximum). Leucine enkephalin was used as reference lock mass calibrant. The acquisition was run in positive mode and data acquisition was carried out using MassLynx 4.1 software (Waters corp., Manchester, UK). The samples were randomized prior to analysis.

### Data pre-processing

Acquired data was processed using Progenesis QI software version 2.3 (Nonlinear Dynamics, Newcastle, UK). Raw data files (centroid data, dead time correction deselected) were uploaded to the software running automatic alignment using the QC samples (pooled samples) to select an alignment reference. The retention time window for peak picking was set to 1.0–11.0 min in positive mode, minimum peak width was fixed to 0.05 min and sensitivity of the peak picking algorithm was set to two. Other parameters were set to default. With these settings Progenesis QI returned 1318 ion features distinguished by retention time and m/z. Ion features with coefficient of variance (CV%) ≤ 30% in the QC samples and m/z > 350 Da were selected and resulted in 439 ion features, which were further analyzed using multivariate data analysis (MVDA). For normalization, the default setting in Progenesis QI *“normalise to all compounds”* was applied. This normalizes all the peak abundances in a sample by the same normalization factor. The normalization factor is calculated on the basis of peaks present in both a reference QC sample and the sample in question. Only peaks that fall within a calculated distance from a median value are included in calculation of the factor. This means that outliers are not included e.g. ions with an abundance of zero for either the normalized or QC sample are not included in the calculation.

### Multivariate data analysis

Principal component analysis (PCA) and orthogonal partial least squares discriminant analysis (OPLS-DA) were applied for MVDA using SIMCA software (version 15, Sartorius Stedim Biotech, Sweden). In the analysis 439 ion features were included. The data was *pareto* scaled prior to modelling. OPLS-DA was performed to determine the most discriminative feature between the reference cell line MCF10A and the individual BC cell line. S-plot visualizes the covariance and the correlation structure between the variables and the predictive score of the predictive component. MCF10A was assigned to the -1 class and each cancer cell line was assigned in class 1. Ion features that were most up- or down-regulated in the specific BC cell line were selected at the cut-off value p(corr) ≥ 0.9 for up-regulated and ≤ -0.9 for the down-regulated ion features, furthermore, ion features with a value of p ≥ 0.14 or ≤ - 0.14 were included (p(corr) > 0.5 or < -0.5).

### Identification of markers

The analytical method allowed detection of lipid species from the classes PC, ether-PC, LPC, PE, ether-PE, LPE, SM, DG and TG in positive mode. The individual lipids were identified according to their mass to charge ratio (m/z), relevant adducts and retention time. The internal standards served as markers for the retention time of lipid subclasses. Possible adducts were assigned to each ion feature in Progenesis QI. GPL and sphingolipids (SL) were identified mainly by identification of the ion adducts [M+H]^+^ and [M+Na]^+^, furthermore, neutral loss of headgroup choline or ethanolamine were used to confirm the identity of PC, SM, and PE, respectively. Glycerolipids (GL) were identified mainly by [H+NH_4_]^+^, [M+H-H_2_O]^+^, and [M+Na]^+^. A combination of an in-house database containing lipid identifier (ID), estimated retention time and neutral mass, online databases e.g. LipidMaps [38, 39] and possible adducts were used in assigning possible lipid ID to each ion feature [40]. A possible ID was assigned to 106 of the included 439 ion features (**S1 Table**). Mass error up to 5 ppm for neutral species was accepted. Data acquisition software Masslynx 4.1 and Targetlynx XS (Waters Corp., Milford, MA, USA) were applied in the estimation of retention times to build up the in-house database.

### Annotation

Lipids from the classes GPL, GL, SL were investigated in this study. The lipids are annotated according to their lipid subclass; PC, LPC, PE, LPE, PG, PI, SM, DG and TG. Individual lipids are characterized by length and composition of the fatty acyls and are annotated as “lipid subclass total carbon number in fatty acyl chains: total number of double bonds” e.g. PC 30:0. Ether-PC and -PE are subdivided into plasmenyl (containing a vinyl group next to the ether bond, also known as plasmalogen) or plasmanyl and will be denoted with the prefix P- or O- respectively [41] e.g. PC P-34:1 or O-34:2. In this paper we do not distinguish between e.g. PC P-34:1 and PC O-34:2. The analytical platform does not allow identification of length of acyl chains and placement of the double bonds, therefore, this is not annotated in this paper.

### Graphical presentation and statistical analysis

RStudio (version 1.1.463, RStudio, Boston, MA, USA) was applied for data analysis, generation of heatmap with hierarchical dendrogram (Euclidean distance and Ward's linkage method) and statistics. GraphPad Prism 5.03 (GraphPad Software, La Jolla, CA, USA) was used for graphical presentation and statistical analysis. Student t-test and two-way ANOVA followed by Bonferroni correction were applied to test statistical difference between mean abundance in BC cell lines and reference cell line for individual lipids.

## Results

### Identification of lipid species by UPLC-QTOF-MS

**Fig 1:**
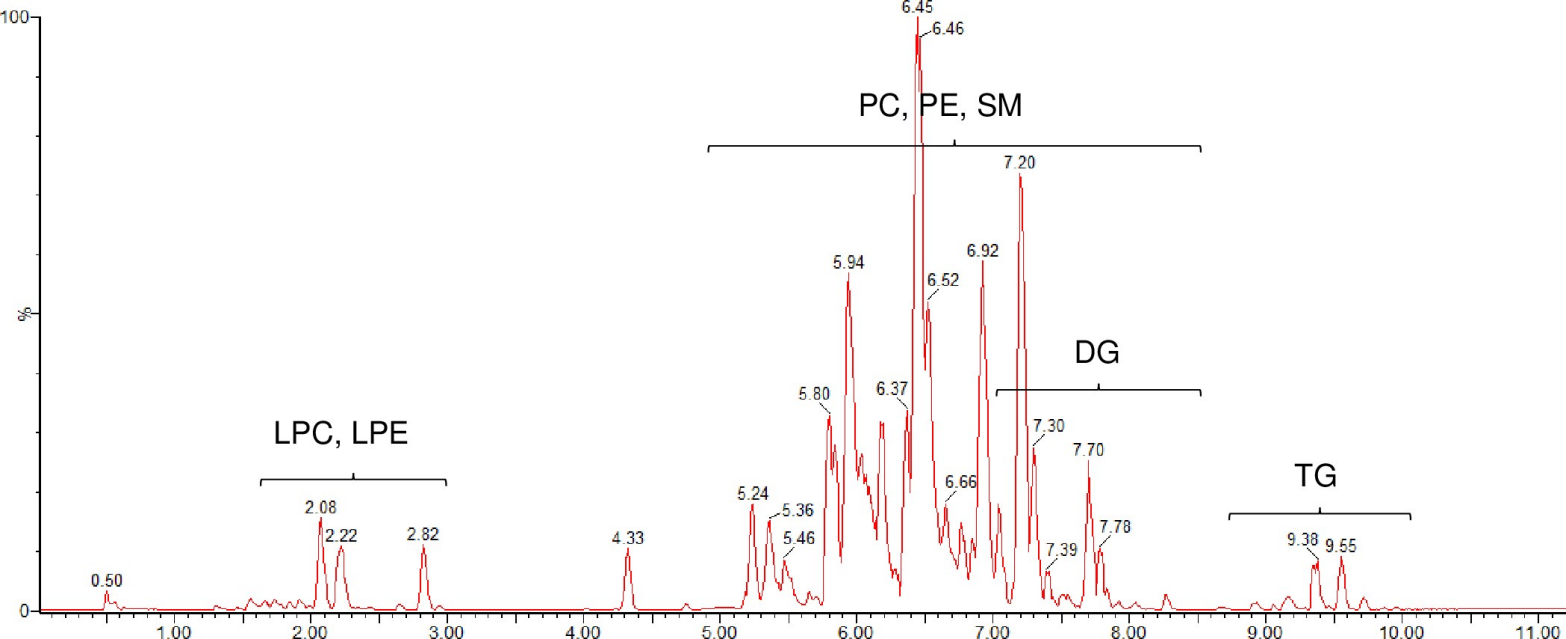
Base peak intensity UPLC-QTOF-MS chromatogram of a QC sample. Retention time windows (min) for investigated lipid subclasses. Positive ionization mode. Time (min) is shown on x-axis and % of the highest peak on y-axis. Peak annotation: LPC–lysophosphatidylcholines, LPE–lysophosphatidylethanolamines, PC–phosphatidylcholines, PE–phosphatidylethanolamines, SM–sphingomyelins, DG–diacylglycerols, TG–triacylglycerols.

In order to evaluate the lipidome of BC cell lines, a fit-for-purpose analytical method is needed for detection and identification of lipids. Here we report a UPLC-QTOF-MS method for analysis of extracted lipids from cultured cell lines. The estimated retention times of individual lipid subclass were 1.5–3.0 min for LPC and LPE, 4.5–8.5 min for PC, PE and SM, 7.0–8.5 min for DG, and 8.5–10 min for TG (**Fig 1**).

### Differences in lipidome between breast cancer cell lines as identified by multivariate data analysis

**Fig 2:**
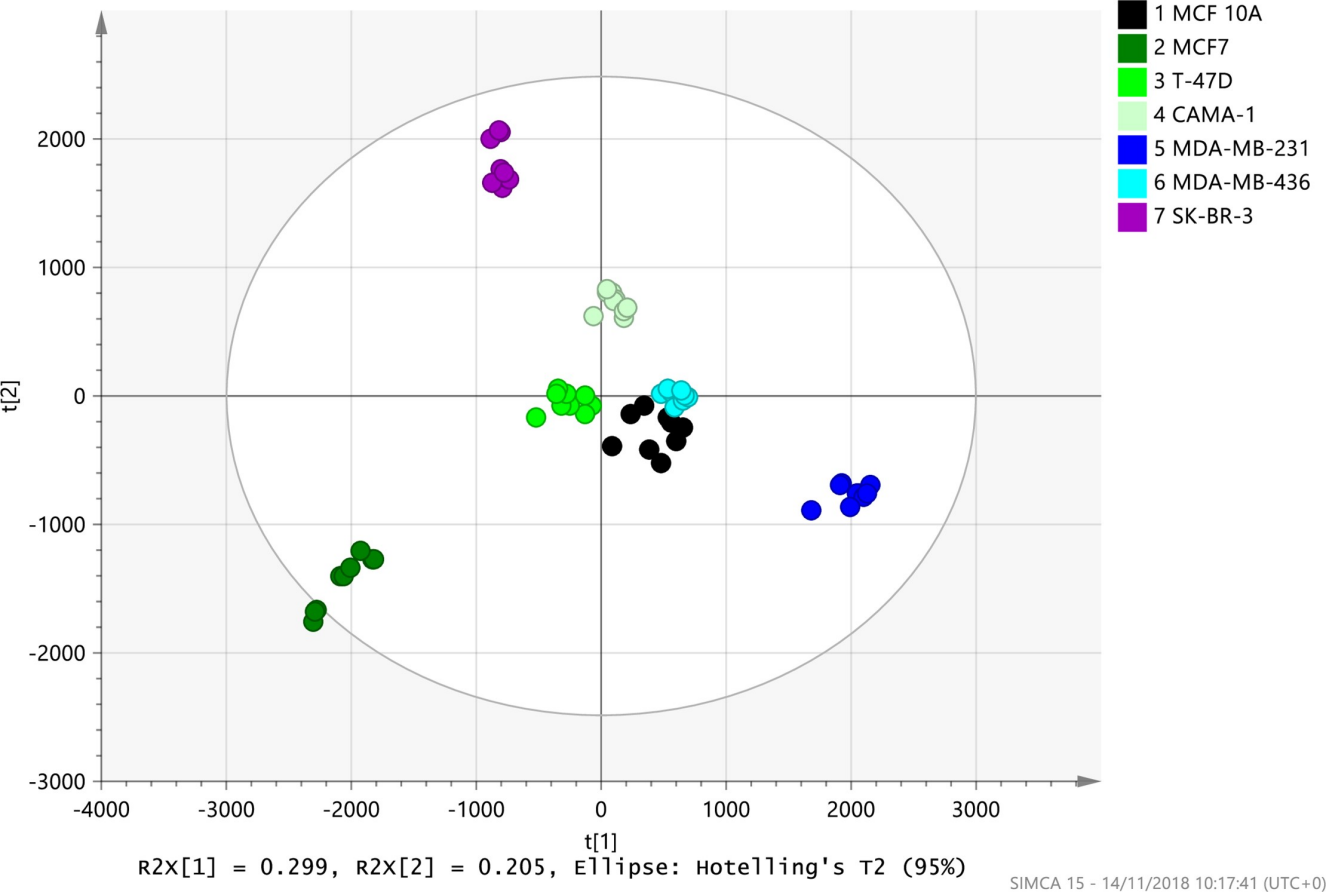
PCA score scatter plot of ion features in breast cancer cell lines and reference cell line. Abundance of 439 ion features normalized to all compounds (CV% ≤ 30%, m/z > 350Da). Pareto scaling and normalization applied to data prior to modelling. Score scatter plot of principal component (PC) 1 and PC 2 after PCA modelling visualizes the differences and similarities in the ion feature profile between the individual cell lines. The white sphere in the model plot represents the Hotelling T2 with 95% confidence. Three biological replicates were analyzed three times with each dot representing one analytical sample.

PCA modelling of 439 ion features detected in Progenesis QI shows that each cell line clustered separate from other cell lines including the reference cell line MCF10A (**Fig 2**). Principal component (PC) 1 on the x-axis explained 29.9% of the variance while PC 2 (y-axis) explained 20.5%. When the QC samples were included, they cluster in the center of the PCA score scatter plot indicating high analytical precision and accuracy (**S1 Fig**). The biological replicates from each individual cell line clustered together indicating low variance within the cell lines. Notably, BC cell lines MCF7, SK-BR-3 and MDA-MB-231 cluster furthest from each other and from the reference cell line, showing the largest difference between components in these cells. The cell lines CAMA-1 and T-47D cluster separate from these BC cell lines and closer to the reference cell line and the MDA-MB-436 cell line. The least difference based on the componential analysis seems to be between MDA-MB-436 and MCF10A.

**Fig 3:**
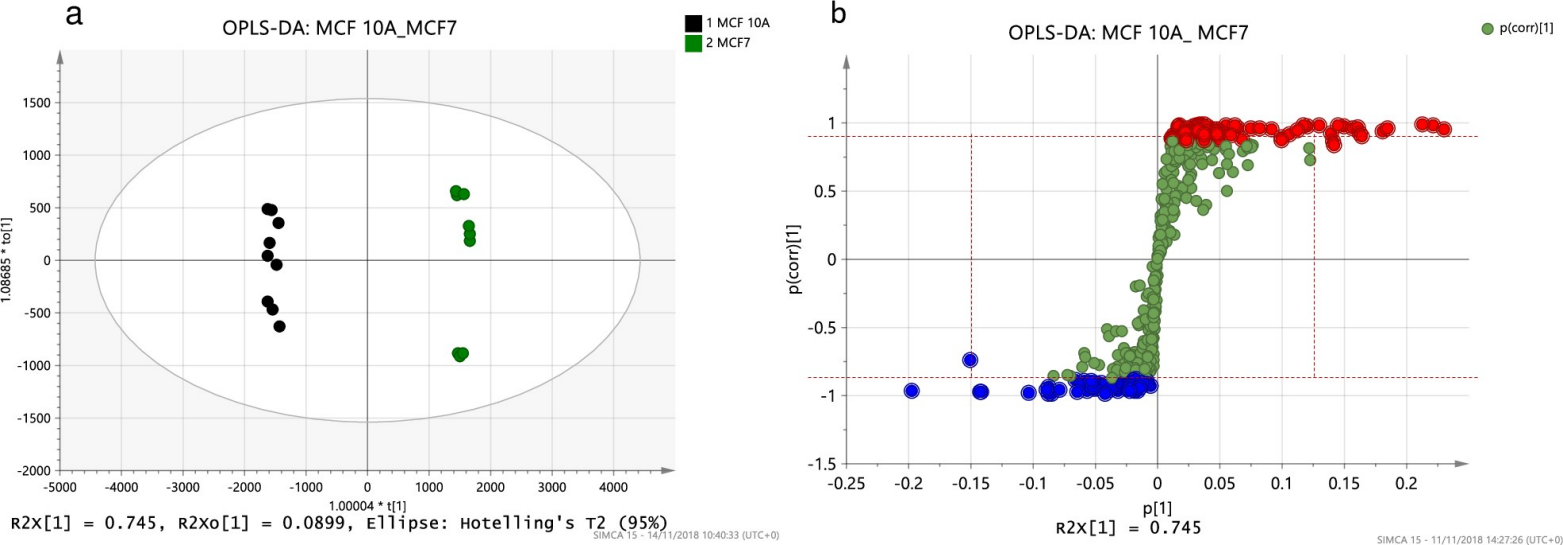
Comparison of MCF7 cell line to reference cell line MCF10A (reference). a) OPLS-DA score scatter plot of MCF7 cell line compared to reference cell line (MCF10A). Scores scatter plot highlights the between-class variance in the predictive component on the x-axis (R2Xo[1]) and the within-class variation in the orthogonal component on the y-axis (to[1]). The black spheres represent MCF10A and green represents MCF7. b) Corresponding S-plot comparing MCF7 to the reference cell line MCF10A. Each green sphere represents an ion feature. The confidence of the ion feature as a discriminant of variance increases with increasing numerical values on the y-axis (-1 or 1) and the size of the contribution increases with increasing numerical values on the x-axis. Ion features selected from S-plots for further identification and processing (cut-off values shown with red dashed lines) are highlighted in red for ion features up-regulated in MCF7 and blue for down-regulated in MCF7. Abundance of 439 ion features normalized to all compounds (CV% ≤ 30%, m/z > 350Da).

An OPLS-DA model for MCF7 cell line compared to the reference cell line (MCF10A) was utilized to uncover the most reliable class discriminating variables (Fig 3, OPLS-DA plots for remaining cell lines can be found in S2 and S3 Figs). Scores scatter plot (**Fig 3A**) highlights the between-cell-lines variance. The explained variance in the predictive component was 74.5% and 8.99% in the orthogonal component, indicating that the variance between the cell lines was larger than between biological and technical replicates of MCF10A or MCF7 cell lines. The selection from the S-plot returned 76 up-regulated (red) and 98 down-regulated (blue) ion features in MCF7 cells compare with MCF10A (**Fig 3B**). The ion features selected across the BC cell lines based on OPLS-DA models included 242 ion features in total and there was substantial overlap between the cell lines.

**Fig 4:**
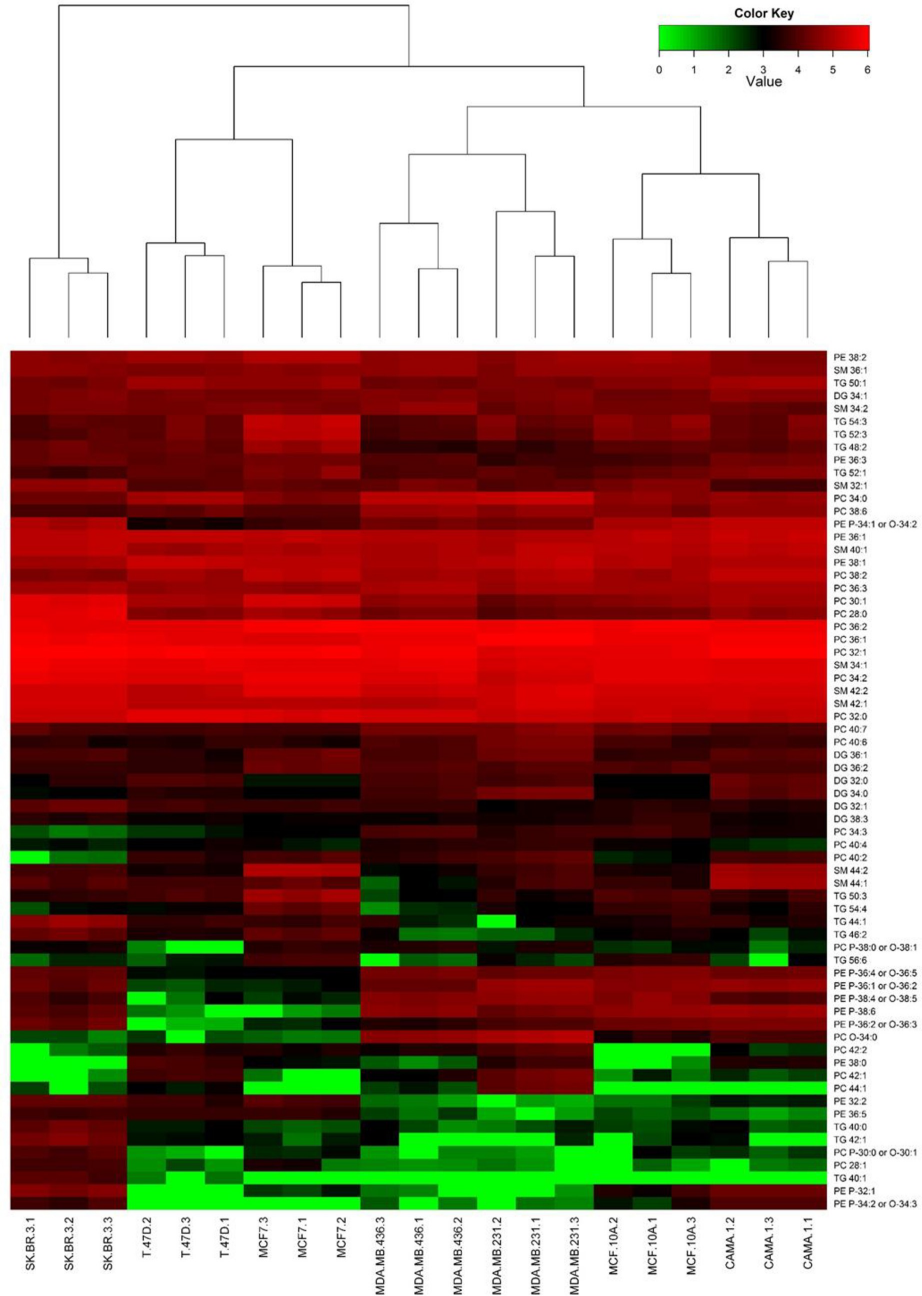
Heatmap including dendrogram of the abundance of selected lipids. Log normalized abundance of selected ion features assigned a possible lipid ID (abundance of lipids ranged from 0 (green) - 10^6^ (red)). The dendrogram shows a hierarchical clustering of the cell lines. Each cell line is represented by three biological replicates (1–3).

A possible lipid ID was assigned to 67 of the 242 ion features belonging to the lipid classes PC, PE, SM, DG and TG. The normalized abundance (log transformed) of these 67 lipid features was included in a heatmap with hierarchical dendrogram (**Fig 4**). The dendrogram showed closest similarity between the biological replicates within each cell line. The cell lines MDA-MB-231 and MDA-MB-436 representing TNBC subtype cluster together and so do cell lines T-47D and MCF7 representing luminal BC subtype. However, high similarity was found between the reference cell line (MCF 10A) and the cell line CAMA-1. These cell lines share similarities with MDA-MB-231 and MDA-MB-436 cell lines. SK-BR-3 cell line was distinguished clearly from the remaining cell lines in the dendrogram.

In the middle of the heatmap, a group of highly abundant lipids of the subclasses SM and PC across all cell lines is seen (lipids with high abundance in all BC cell lines including the reference cell line, see **S2 Table**). These are mainly C-32 to C-36 lipids, except for two C-42 SM. A group of ether-PE and -PC (C-34 to C-38) had a very low abundance (green color, **Fig 4**) in MCF7 and T-47D compared to the other cell lines. Some of the TG grouped together and showed a high abundance in either MCF7 or SK-BR-3. Otherwise, the heatmap shows that differences between BC cell lines were related to individual lipids belonging to subclasses PC, PE, SM and DG, which will be elaborated below.

### Specific lipids are up- or down-regulated in individual breast cancer cell lines

**Fig 5:**
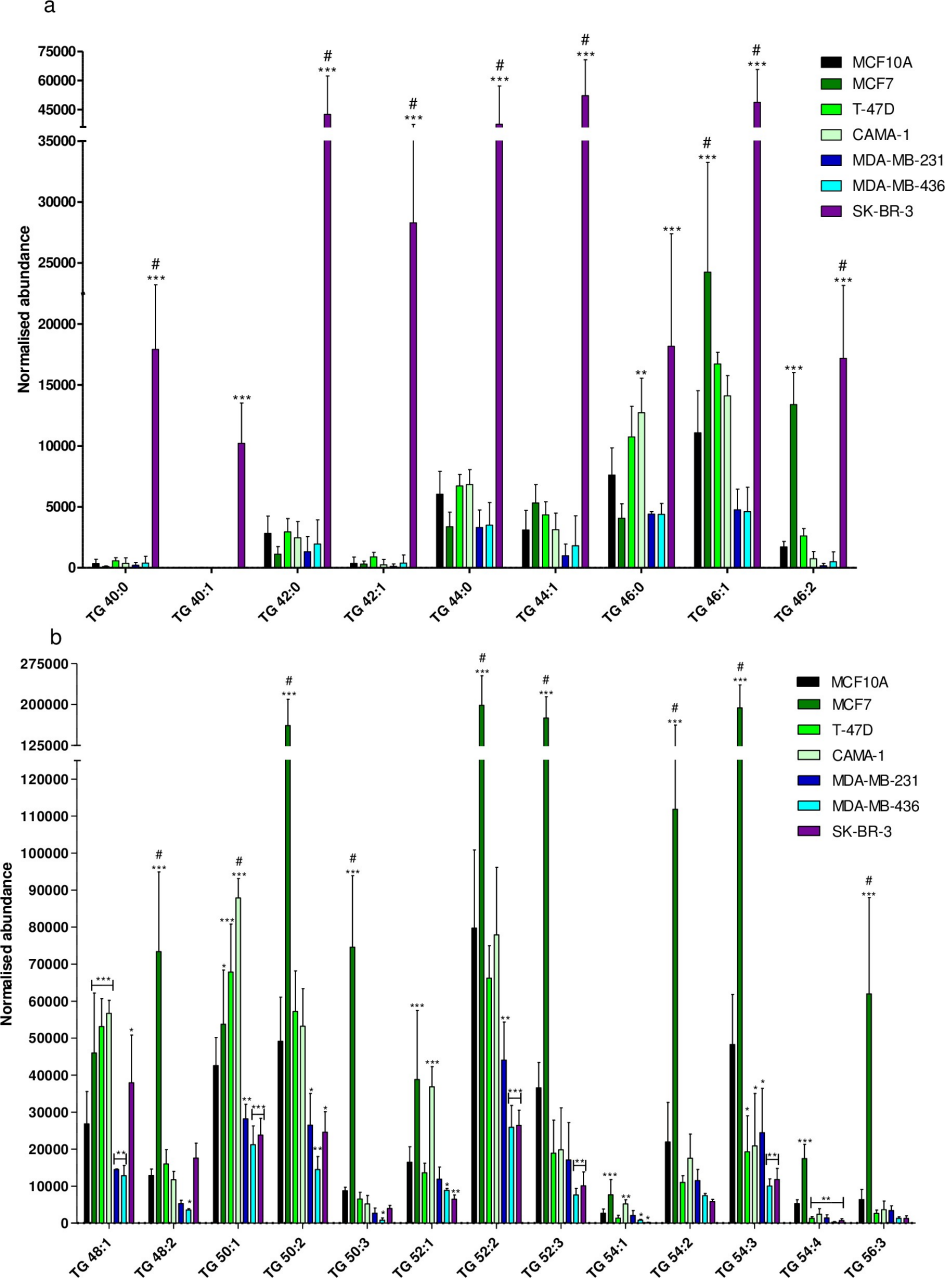
Normalized abundance of selected triacylglycerols (TG). a) C-40 to C-46 TG and b) C-48 to C-56 TG. Bars represent mean abundance of three biological replicates, error bars represent standard deviation (SD). The normalized abundance is shown on the y-axis. Statistically significant increase of TG normalized abundance compared to MCF10A is indicated by * (p< 0.05), ** (p<0.01), *** (p<0.001), insignificant changes are unmarked. # indicates significant up-regulation after adjusting for multiple testing using two-way ANOVA followed by Bonferroni correction (p<0.05).

In two BC cell lines, SK-BR-3 and MCF7, TG were found to be especially abundant (**Fig 5**). In SK-BR-3 cells, TG ≤ C-46 with saturated or monounsaturated FA (MUFA) in side chains (TG 40:0, TG 40:1, TG 42:0, TG 42:1, TG 44:0, TG 44:1, TG 46:1 and TG 46:2) were found to be up-regulated compared with all other cell lines (**Fig 5A**). MCF7 cells showed a significantly higher abundance of TG 46:1 and 46:2, and C-48 to C-56 TG (**Fig 5B**). The abundance was remarkably high in TG with di- or tri-unsaturated FAs (TG 48:2, TG 50:2, TG 50:3, TG 52:2, TG 52:3, TG 54:2 and TG 54:3) when compared with the other cell lines (**Fig 5B**). TG ≥C-50 were generally down-regulated in SK-BR-3 and in one or both of the MDA-MB-436 or MDA-MB-231 TNBC cell lines (**Fig 5B**).

**Fig 6:**
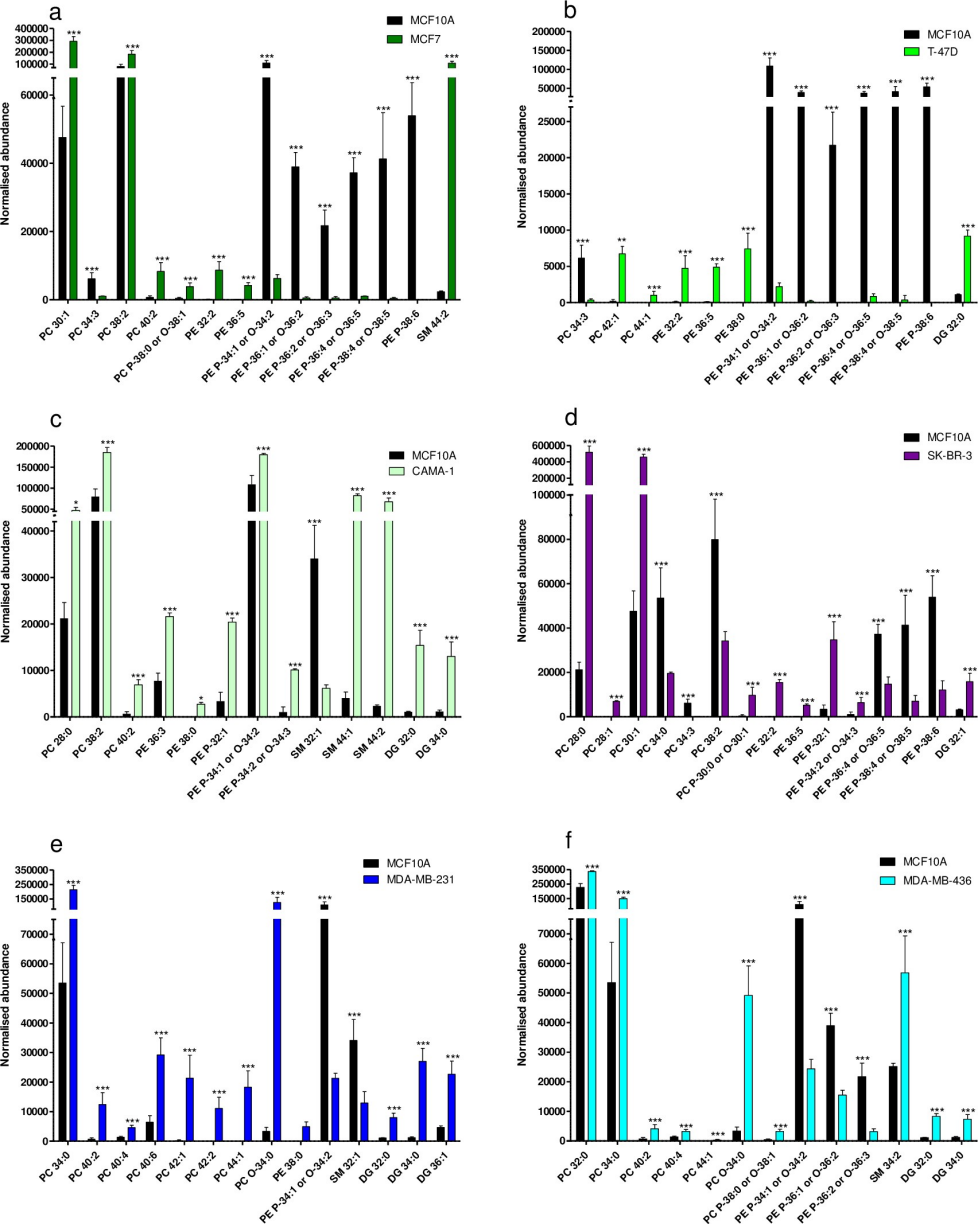
Normalized abundance of most significantly up- or down-regulated lipids in each cell line. a) MCF7, a) T-47D, c) CAMA-1, d) SK-BR-3, e) MDA-MB-231, and f) MDA-MB-436 compared to the reference cell line MCF10A cell line. Bars represent mean abundance of three biological replicates, error bars represent SD. Normalized abundance is shown on the y-axis. Statistically significant up- or down-regulation in normalized abundance compared to MCF10A is indicated by * (p< 0.05), ** (p<0.01), *** (p<0.001).

The heatmap (**Fig 4**) showed that the trends for the lipid subclasses PC, PE, SM and DG, were not as clear as for the TG. However, even though many of the identified lipids were present in multiple BC cell lines, a differences were found in up- or down-regulation of specific lipids between the cell lines (**Fig 6**). PC with a low number of total carbons and saturated FA or MUFA in side chains PC 28:0, PC 28:1, or PC 30:1 were up-regulated in MCF7, CAMA-1 and/or SK-BR-3, but not in MDA-MB-231 or MDA-MB-436 (**Fig 6A–6D**). A shared tendency between MCF7 and T-47D was a significant down-regulation in ether-PE (**Fig 6A** and **6B**), whereas in CAMA-1 PE P-34:1/O-34:2, PE P-34:2/O-34:3 and PE P-32:1 were significantly up-regulated (**Fig 6C**). In SK-BR-3 PE P-32:1 and PE P-34:2/O-34:3 were also up-regulated, however, PE P-36:4 or O-36:5 and PE P-38:4 or O-38:5 were down-regulated (**Fig 6D**). The luminal cell lines and SK-BR-3 cells shared features in significant up-regulation of specific PE (PE 32:2 and PE 36:5 were up-regulated in MCF7, T-47D, SK-BR3 and PE 36:3 in CAMA-1). In CAMA-1 and T-47D PE 38:0 was significantly up-regulated. SM 44:1 and SM 44:2 were highly up-regulated in CAMA-1 cells, which was not the case for SM of shorter chain lengths, thus SM 32:1 was significantly down-regulated (**Fig 6C**). SM 44:2 was also up-regulated in MCF7 (**Fig 6A** and **6B**). Some shared features between MDA-MB-231 and MDA-MB-436 cell lines were a significant up-regulation of PC 34:0 and PC O-34:0 for both cell lines and PE P-34:1/O-34:2 down-regulation (**Fig 6E** and **6F**). In these cell lines, there was a tendency for up-regulation of PC ≥C-40 compared to the reference. Some of these PC were also upregulated in MCF7 (PC 40:2) and T-47D (PC 42:1 and 44:1). SM 32:1 was significantly down-regulated in MDA-MB-231, whereas SM 34:2 was significantly up-regulated in MDA-MB-436. In both MDA-MB-231 and MDA-MB-436 cell lines, DG 32:0 and DG 34:0 were significantly up-regulated (**Fig 6E** and **6F**) which was similarly observed for CAMA-1 and T-47D cell lines (**Fig 6B** and **6C**). Six LPC were identified (LPC 14:0, LPC 16:0 LPC 16:1, LPC 18:0, LPC 18:1 and LPC 18:2) with the abundance being largely similar to the reference cell line (S4 Fig). MCF7 showed the most significant differences in LPC, with up-regulation in all LPC except for LPC 18:0. LPC 16:0 and LPC 18:0 was up-regulated in T-47D and MDA-MB-436, but for MDA-MB-231 only LPC 18:0 was upregulated. Among LPE only LPE 20:1 was identified in the dataset with similar abundance across all cell lines.

## Discussion

The PCA scatter plot clearly defined clustering of each BC cell line indicating that the ion features detected are sufficient to distinguish between the cell lines analyzed. Clustering of the cell lines in a hierarchical dendrogram based on lipids from the subclasses PC, PE, SM, DG and TG, showed the relative similarities between the cell lines. The closest similarities were observed between cell lines MDA-MB-231 and MDA-MB-436 which are both TNBC tumor subtype. Furthermore, the luminal cell lines MCF7 and T-47D were similarly clustered in the dendrogram. These similarities found in the dendrogram are reflected in the PCA by clustering in the same quadrant (MDA-MB-231 and MDA-MB-436 in 4^th^ quadrant and MCF7 and T-47D in 3^rd^ quadrant). CAMA-1 was, however, more related to the reference cell line than the other luminal cell lines based on the dendrogram and in the PCA plot it was in quadrant 1 close to the reference cell line, suggesting similarities in the lipidome of these cells. Conflicting results exist in the literature regarding the PgR status of CAMA-1 which is reported to be positive or negative in different studies. Furthermore, the *TP53* status is different for the three luminal cell lines with T-47D expressing mutated protein [6, 27] and *TP53* is known to regulate lipid metabolism in cancer [42]. This indicates within-group differences in the lipidome of cells representing the luminal subtype and suggests that CAMA-1 should not necessarily be classified as the same luminal subtype as MCF7 and T-47D. The cell line that was most dissimilar from the other cell lines based on both PCA and dendrogram was SK-BR-3 cell line. SK-BR-3 is a representative of HER2 overexpressing tumor subtype that is known to have increased FASN activity resulting in increased lipid synthesis and changes in the lipidome [43, 44]. The clustering verified that BC subtypes could be distinguished based on the lipidome and suggests that there is within-subclass variation in the lipidome.

### The potential of triacylglycerols to distinguish between BC cell lines

TG stored in the cell as lipid droplets represent a reservoir of FAs ready to undergo β-oxidation. We detected significant changes in TG particularly in SK-BR-3 and MCF7 cells. The perilipins (PLIN 1–5) are generally involved in regulation of TG storage. PLIN1 suppresses the hydrolysis of TG from lipid droplets and is generally down-regulated in BC with the lowest expression in TNBC [11, 17]. In contrast, HER2 tumors have a higher expression of PLIN1 [11], which could explain our finding of increased abundance of TG ≤ C-46 in SK-BR-3. In breast tumor tissue, where lipids were investigated using evaporating ionization MS, TG ≥ C-50 were significantly decreased [45]. The investigated tissues were mainly ER-positive and HER2-negative [45], similar to luminal subtypes. These results are not in line with our findings, indicating a significant increase in TG ≥ C-48 in MCF7. In another study TG were mainly unaltered or down-regulated in BC tissue when compared with normal, with no significant difference based on ER or HER2 status. However, there was a tendency for up-regulation of TG in the PgR-negative compared with PgR-positive tumor tissues [46]. Recently Paul et al. showed that in malignant BC tissue the total amount of TG measured by NMR was decreased compared with benign BC tissue [47], subtype not specified. Interestingly, in several studies the plasma levels of TG were elevated in BC patients [48–50]; however, the BC subtypes were not reported in these studies. Mammary glands are known to produce TG containing shorter chain FA [51] and in peroxisomes, β-oxidation in peroxisomes can produce chain-shortened acyl-CoAs that can be incorporated into lipids [52]. β-oxidation is mediated by acyl-CoA oxidases (ACOX 1–2) and the protein level of ACOX-1 was shown to be increased in HER2-cell line and tumor tissue compared with other BC subtypes [11, 17]. Therefore, increased β-oxidation in HER2-overexpressing cells could explain the difference in TG between SK-BR-3 and other cell lines. The trend of up-regulation of TG ≤ C-50 and down-regulation of TG ≥ C-50 in SK-BR-3 is also true for other lipid subclasses: PC and PE (≤ C-32) were up-regulated in SK-BR-3 and PC ≥ C-34 with longer chain lengths were significantly down-regulated. Furthermore, ether-PC (C-30) and two ether-PE (C-32 and C-34) were up-regulated while three ether-PE (C-36 and C-38) with multiple double bonds were down-regulated. Hilvo et al. and Kang et al. reported a significantly increased levels of PC 30:0 in ER- and PgR-negative and HER2 tumor subtypes compared with the corresponding ER- and PgR-positive and HER2-negative subtypes [21, 46] in line with the findings for SK-BR-3 in our study. Overall, our results suggest that mainly short-chain moderately unsaturated FAs are incorporated in GPL and TG in SK-BR-3 cells.

### Highly abundant lipids contain C-16 and C-18 fatty acyl chains

In our study, the most abundant PC and PE had a composition of C-32 to C-36 saturated or moderately unsaturated (**S2 Table**), indicating that the side chains were C-16:0, C-16:1, C-18:0, or C-18:0 FA which are predominantly synthesized via FASN [16, 20] and are the most prevalent constituents of lipids in mammalian cell membranes [10, 53]. Other studies of human BC cell lines (T-47D and MDA-MB-231 and non-cancerous MCF10A) similarly reported the highest abundance of PC and PE of similar compositions in all cell lines and cancer tissue [21, 24, 25, 46]. In these studies there were variations in these abundant lipids between BC and reference cell lines, however, different lipids were found to be upregulated [24, 25, 54]. In our study, the abundance of these lipids varied up to 2.9 fold between individual BC cell lines, whereas we generally found the highest fold differences in abundance compared to reference for PC < C-32 or > C-36 (**Fig 6**). Therefore, these abundant lipids will not be considered further for distinguishing BC subtypes in this paper.

### Increased phosphatidylcholine synthesis in triple negative breast cancer cells

In our study particularly DG 32:0 and DG 34:0 were significantly more abundant in BC cell lines than the reference cell line, especially, in the TNBC cell lines and CAMA-1. Increased plasma levels of DG (C-32 to C-38) have also been reported in BC patients [49, 50]. DG are precursors to GPL which are synthesized *de novo*, and to TG stored in droplets [15, 55]. Lipin-1 (LPIN1) catalyzes the conversion of phosphatic acid to DG and is involved in the accumulation of lipids in droplets [17, 55]. LPIN1 has been shown to be overexpressed in TNBC, be lower in HER2 and lowest in luminal subtypes [17]. In combination with elevated DG levels and decreased level of TG in TNBC this may indicate an increased production of GPL in the TNBC cell lines. In the TNBC cell lines the PC that showed the most significant difference from the reference cell line were PC ≥ C-40. In plasma and tissue from BC patients the levels of PC C-30 to C-38 were increased [46, 49, 56, 57]. However, above PC 40:0 the literature offers conflicting information. In accordance with our study, PC 40:4 and PC 40:6 were significantly increased in BC tumor tissue with a higher abundance in ER-negative than ER-positive tumor subtype [46]. PC 40:6 was increased in plasma whereas PC 40:2 and PC 40:4 were decreased (subtype of tumors not reported) [57]. Elongation of saturated FAs is mediated by ELOVL 1–7 [58]. ELOVL 1 and 6 mRNA levels were up-regulated in TNBC when compared with the luminal A subtype and up-regulated in both compared with normal tissue [59]. This suggests that the increase in PC ≥ C-40 in TNBC cell lines may be explained by an increased production of long-chain FAs. Some PC (≤ C-30) with saturated FAs or MUFAs in acyl chains were up-regulated in MCF7, CAMA-1 and/or SK-BR-3, but not TNBC cell lines, which also correlates with the expression data for ELOVL. Collectively, it seems that an increased level of PC ≥ C-40 may be related to the TNBC subtype.

### Ether-glycerophospholipids are down-regulated in ER- and PgR-positive cancer cells

Ether-GPL are abundant in biological membranes, they play a role as second messengers, in differentiation and storage of long-chain PUFAs, they are abundant in lipid raft microdomains and they are suggested to protect cells from reactive oxygen species [60]. The biosynthesis of these lipid species is, however, not fully understood [61]. To our knowledge, the enzymes involved have not been investigated in relation to BC. The most abundantly produced ether-GPL are esterified with PUFA (i.e. C-22:6 or C-20:4) at the sn-2 position of the glycerol backbone, whereas sn-1 position is saturated or moderately unsaturated with a length of C-16 or C-18 [60]. These combinations result in C-36 to C-40 with 4 to 6 double bonds, which corresponds to the most reported ether-PE in the literature, in combination with ether-PE containing a total of 34 carbons [24, 25]. In our study, the abundances of ether-PE were generally lower in the luminal cell lines MCF7 and T-47D. A similar trend was reported in MCF7 and T-47D by Katz-Brull et al. [23] and by Sterin et al. in MCF7 and two other luminal cell lines [22]. The general trend reported by Cifkóva et al. was that P-PC and P-PE were similar or down-regulated in BC cell lines when compared with the reference [25]. Interestingly, we show here a higher abundance of three PE in SK-BR-3 and CAMA-1 when compared with the reference cell line, which may indicate that the ether-PE down-regulation is related to the ER- and PgR-positive status subtypes (contradicting reports of CAMA-1 PgR status [27]). Doria et al. showed highest relative abundance of PC O-34:1 and PC O-36:1 in MDA-MB-231 when compared with reference and T-47D [24]. In our study the same trend was seen for PC O-34:0 which was significantly up-regulated only in the TNBC cell lines. Tissue samples from benign hyperplastic-dysplastic and malignant BC showed an increase in ether-PE and ether-PC when compared with normal tissue [62]. BC subtype was not reported. In support of our findings, Hilvo et al. reported significantly lower levels of ether-PE in ER-positive than ER-negative cancer subtypes [46]. Based on our results ether-PE may be down-regulated in ER- and PgR-positive compared to ER- and PgR-negative BC subtype.

### Limitations of the study design

This study is based only on cell lines. This has the advantage of using well-defined material that has also been used in other lipidomic studies, thus offering the opportunity for comparison. A major drawback is that each cell line can only reflect the individual tissue of origin and caution is needed in generalizing findings. Furthermore, these cell lines have been in culture for a long time and have adapted to *in-vitro* conditions. The cell lines represent different defined subtypes of breast cancer, but only one cell line was derived from a HER2-positive cancer. The comprehensive comparison of Jiang *et al*. of 68 breast cancer cell lines and primary breast cancer tissue revealed strong correlations particularly for mRNA expression but weaker for genomic profiles and protein expression. In a combined correlation score the T47-D, CAMA-1 and SK-BR-3 cell lines scored high, but MCF-7 and MDA-MB-231 and MDA-MB-436 had a lower score [63]. The conventional 2D cultures are a further limitation. Cell shape and microenvironment influence cellular lipid content and composition [64] and 2D cultures do not bring out site-specific transcriptomic profiles of metastases [65]. The added serum is the main source of lipids on the culture medium, and this was the same for all cell lines.

## Conclusion

In this work, we describe the most significant differences in the lipidome between individual BC cell lines and a reference cell line. Firstly, we established the overall similarities in the lipidome of the cell lines based on PCA and hierarchical dendrogram. The most similar cell lines belonged to the same subtypes; the TNBC cell lines (MDA-MB-231 and MDA-MB-436) and the two luminal cell lines (T-47D and MCF7). The luminal cell line CAMA-1 did not share strong similarity with the other luminal cell lines revealing within-subtype variations in the lipidome. The cell line that differed most based on the lipidome from the other cell lines was the HER2-overexpressing, SK-BR-3 cell line. Some general trends in the lipidome may be useful for distinguishing between BC tumor subtypes in clinical samples, particularly if applied to plasma samples. Most interestingly, we suggest that ER- and PgR-positive tumor subtypes can be identified by a significantly increased abundance of TG ≥ C-48 with moderate or multiple unsaturated FA chains, which in contrast are significantly down-regulated in ER- and PgR-negative subtypes (HER2 and TNBC). Furthermore, ether-PE, especially those containing the most abundant fatty acyls C-16, C-18 and C-22:6 or C-20:4 may be down-regulated in ER- and PgR-positive subtypes and to a lesser degree in ER- and PgR-negative subtypes. Therefore, these PE could be of particular interest for distinguishing between ER- and PgR-positive and -negative subtypes. Furthermore, we suggest that the HER2-overexpressing tumor subtype is characterized by elevated levels of TG, PC and PE containing saturated FA or MUFA ≤ C-16 in the side-chains. Significantly increased abundance of PC ≥ C-40 may be useful for identification of the TNBC subtype. In addition, differences were detected in lipidomes within these previously defined subtypes. We conclude that subtypes defined by the transcriptome are indeed reflected in the lipidome which may be used to define further subdivision within the BC subtypes.

### Closing remarks

The cells analyzed in this study are grown under controlled conditions. They represent good and reasonably stable models of clinical phenotypes and reflect to some extent molecular characteristics of primary tumors [33, 63]. The biological variation and complexity of lipid metabolism in clinical samples is expected to be greater. Therefore, the trends and specific up- or down-regulations presented here cannot be directly extrapolated to clinical plasma or tissue samples. Clinical investigations of the lipidome in healthy individuals show that the plasma levels of lipids exhibit large inter- and intra-individual variation which follow a circadian rhythm and fluctuate over time [66]. Even comparison with other studies investigating the same or similar BC cell lines sometimes offered dissimilar results which could have various reasons, including changes acquired with time and differences in culture conditions and sample preparation methods. Performing and interpreting lipidomic analysis, including sample preparation, analytical setup, lipid identification and data interpretation is complex and challenging [67]. Furthermore, the availability of essential lipids as building blocks for e.g. GPLs is likely to be quite different *in vitro* and *in vivo*. The potential of lipidomics as a diagnostic tool needs to be investigated further, and the recently published study of Santoro *et al*. implies that this is a promising approach [68]. The results presented here need, firstly, to be confirmed by a targeted MS approach. Secondly, the similarity of the lipidome of the *in vitro* cultured BC cell lines and actual BC tumor subtypes needs to be confirmed in clinical samples.

## Supporting information

S1 Table Normalized abundance of 106 identified lipids from the 439 ion features included in the PCA. (XLSX)

S2 Table High abundant lipids expressed in all cell lines. (DOCX)

S1 Fig PCA score scatter plot of ion features in breast cancer cell lines, reference cell line and QC samples. Abundance of 439 ion features normalized to all compounds (CV% ≤ 30%, m/z > 350Da). Pareto scaling and normalization applied to data prior to modelling. Score scatter plot of principal component (PC) 1 and PC 2 after PCA modelling visualizes the differences and similarities in the ion feature profile between the individual cell lines. The white sphere in the model plot represents the Hotelling T2 with 95% confidence. Three biological replicates were analyzed three times with each dot representing one analytical sample and QC samples consisted of 11 injections throughout the analytical batch.
(TIF)

S2 Fig Comparison of T-47D, CAMA-1, and SK-BR-3 cell lines to reference cell line MCF10A. a) OPLS-DA score scatter plot of T-47D cell line compared to reference cell line the reference cell line MCF10A. b) Corresponding S-plot comparing T-47D to the reference cell line MCF10A. c) OPLS-DA score scatter plot of CAMA-1 cell line compared to reference cell line MCF10A. The black spheres represent MCF10A and green represents CAMA-1. d) Corresponding S-plot comparing CAMA-1 to the reference cell line MCF10A. e) OPLS-DA score scatter plot of SK-BR-3 cell line compared to reference cell line MCF10A. The black spheres represent MCF10A and green represents SK-BR-3. f) Corresponding S-plot comparing SK-BR-3 to the reference cell line MCF10A. Scores scatter plots highlight the between class variance in the predictive component on the x-axis (R2Xo [1]) and the within class variation in the orthogonal component on the y-axis (to[1]). In OPLS-DA each green sphere represents an ion feature. The confidence of the ion feature as a discriminant of variance increases with increasing numerical values on the y-axis (-1 or 1) and the size of the contribution increases with increasing numerical values on the x-axis. Ion features selected from S-plots for further identification and processing (cut-off values shown with red dashed lines) are highlighted in red for ion features up-regulated in BC cell line and blue for down-regulated in BC cell line compared to reference. Abundance of 439 ion features normalised to all compounds (CV% ≤ 30%, m/z > 350Da).
(TIF)

S3 Fig Comparison of MDA-MB-231 and MDA-MB-436 cell lines to reference cell line MCF10A. a) OPLS-DA score scatter plot of MDA-MB-231 cell line compared to reference cell line MCF10A. The black spheres represent MCF10A and green represents MDA-MB-231. b) Corresponding S-plot comparing MDA-MB-231 to the reference cell line MCF10A. c) OPLS-DA score scatter plot of MDA-MB-436 cell line compared to reference cell line MCF10A. The black spheres represent MCF10A and green represents MDA-MB-436. d) Corresponding S-plot comparing MDA-MB-436 to the reference cell line MCF10A. Scores scatter plots highlight the between class variance in the predictive component on the x-axis (R2Xo [1]) and the within class variation in the orthogonal component on the y-axis (to[1]). In OPLS-DA each green sphere represents an ion feature. The confidence of the ion feature as a discriminant of variance increases with increasing numerical values on the y-axis (-1 or 1) and the size of the contribution increases with increasing numerical values on the x-axis. Ion features selected from S-plots for further identification and processing (cut-off values shown with red dashed lines) are highlighted in red for ion features up-regulated in BC cell line and blue for down-regulated in BC cell line compared to reference. Abundance of 439 ion features normalised to all compounds (CV% ≤ 30%, m/z > 350Da).
(TIF)

S4 Fig Normalised abundance of identified LPCs. Bars represent mean abundance of three biological replicates, error bars represent SD. Normalised abundance is shown on the y-axis. Statistically significant up- or down-regulation in normalised abundance compared to MCF10A is indicated by * (p< 0.05), ** (p<0.01), *** (p<0.001), insignificant changes are unmarked.
(TIF)
