# Supplementary material for: Lipidomic study of cell lines reveals differences between breast cancer subtypes
Source: PLoS One. 2020 Apr 14;15(4):e0231289. doi: 10.1371/journal.pone.0231289 (PMC7156077; doi:10.1371/journal.pone.0231289)
Supplement: S2 Table — (DOCX) [file pone.0231289.s002.docx]

**S1 Table.** **High abundant lipids expressed in all cell lines.**

| Possible ID | m/z | Retention time (min) | Neutral mass (Da) | Exact mass | Adducts | Mass Error (ppm) | Max Fold change |
| --- | --- | --- | --- | --- | --- | --- | --- |
| PC 32:0 | 734.568 | 6.3685 | 733.562 | 733.560 | M+H, M+Na | -2.49 | 2.8 |
| PC 32:1 | 732.551 | 5.94 |  | 731.547 | M+H | -3.41 | 2.2 |
| PC 34:1 | 760.580 | 6.45 | 759.573 | 759.578 | M+H, M+Na | -6.50 | 1.2 |
| PC 34:2 | 758.568 | 6.11 | 757.560 | 757.572 | M+H, M+Na | -2.33 | 2.4 |
| PC 36:1 | 788.613 | 6.92 | 787.605 | 787.609 | M+H, M+Na | -4.71 | 2.6 |
| PC 36:2 | 786.597 | 6.53 | 785.590 | 785.594 | M+H, M+Na | -4.37 | 1.6 |
| PE 34:1 | 718.542 | 6.59 | 717.535 | 717.531 | M+H, M+Na | 6.38 | 2.0 |
| PE 36:1 | 746.570 | 7.04 | 745.563 | 745.562 | M+H, M+Na | 0.58 | 2.0 |
| PE 36:2 | 744.561 | 6.66 | 743.553 | 743.547 | M+H, M+Na | 9.09 | 2.9 |
| PE 38:1 | 774.599 | 6.70 |  | 773.593 | M+H | -1.63 | 2.5 |
| SM 34:1 | 703.574 | 5.80 |  | 702.568 | M+H | -1.68 | 1.9 |
| SM 40:1 | 787.668 | 7.30 | 786.660 | 786.662 | M+H, M+Na | -1.30 | 2.2 |
| SM 42:1 | 815.698 | 7.70 | 814.690 | 814.693 | M+H, M+Na | -2.90 | 2.3 |
| SM 42:2 | 813.682 | 7.30 | 812.675 | 812.677 | M+H, M+Na | -3.07 | 2.8 |

Max fold change represents the maximum fold change between any cell lines. 10 ppm difference mass error was accepted for highly abundant lipids.
