# Supplementary figures and images for: Lipidomic study of cell lines reveals differences between breast cancer subtypes
Source: PLoS One. 2020 Apr 14;15(4):e0231289. doi: 10.1371/journal.pone.0231289 (PMC7156077; doi:10.1371/journal.pone.0231289)

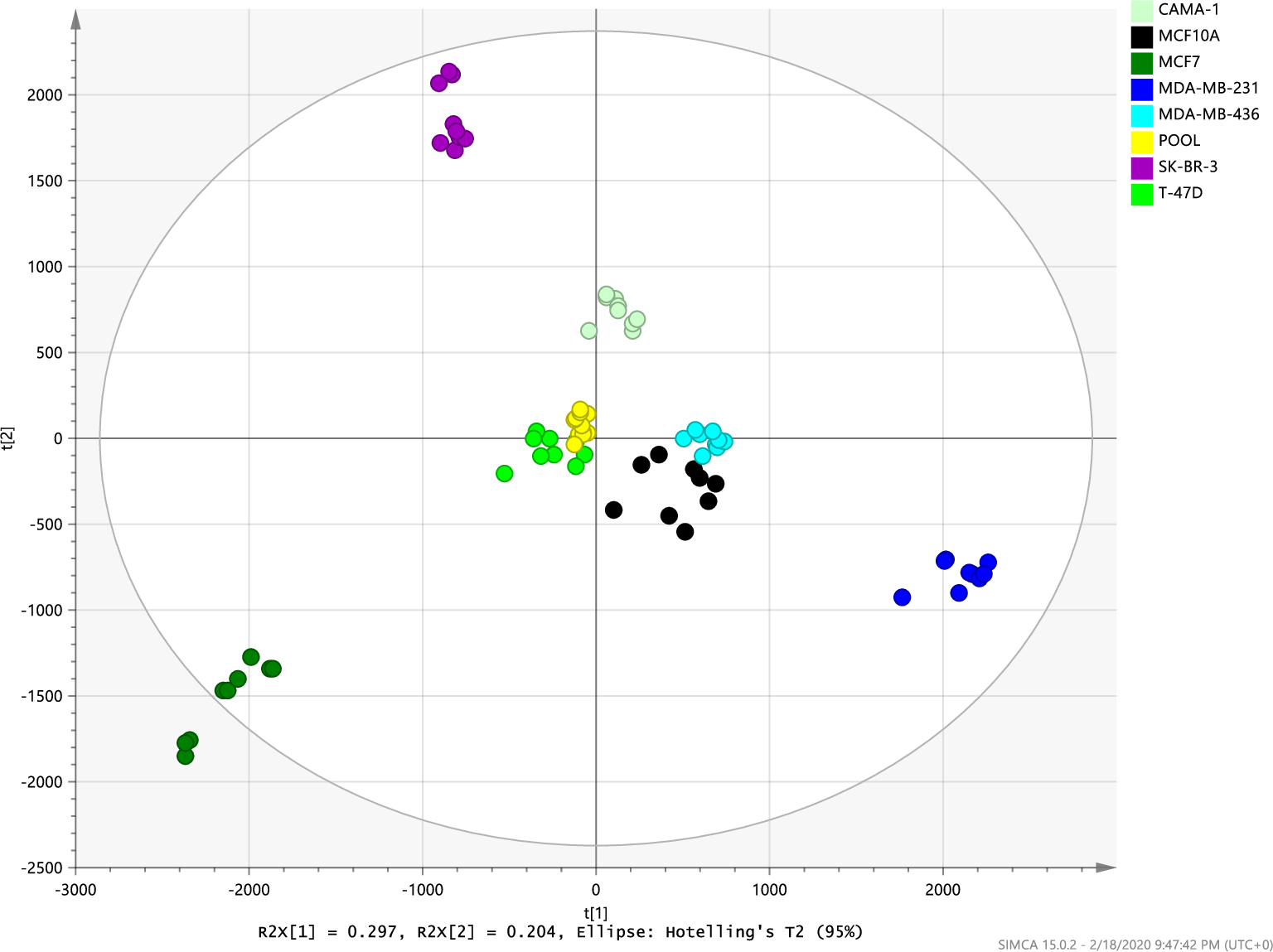

Supplement: S1 Fig — Abundance of 439 ion features normalized to all compounds (CV% ≤ 30%, m/z > 350Da). Pareto scaling and normalization applied to data prior to modelling. Score scatter plot of principal component (PC) 1 and PC 2 after PCA modelling visualizes the differences and similarities in the ion feature profile between the individual cell lines. The white sphere in the model plot represents the Hotelling T2 with 95% confidence. Three biological replicates were analyzed three times with each dot representing one analytical sample and QC samples consisted of 11 injections throughout the analytical batch. (TIF) [file pone.0231289.s003.tif]

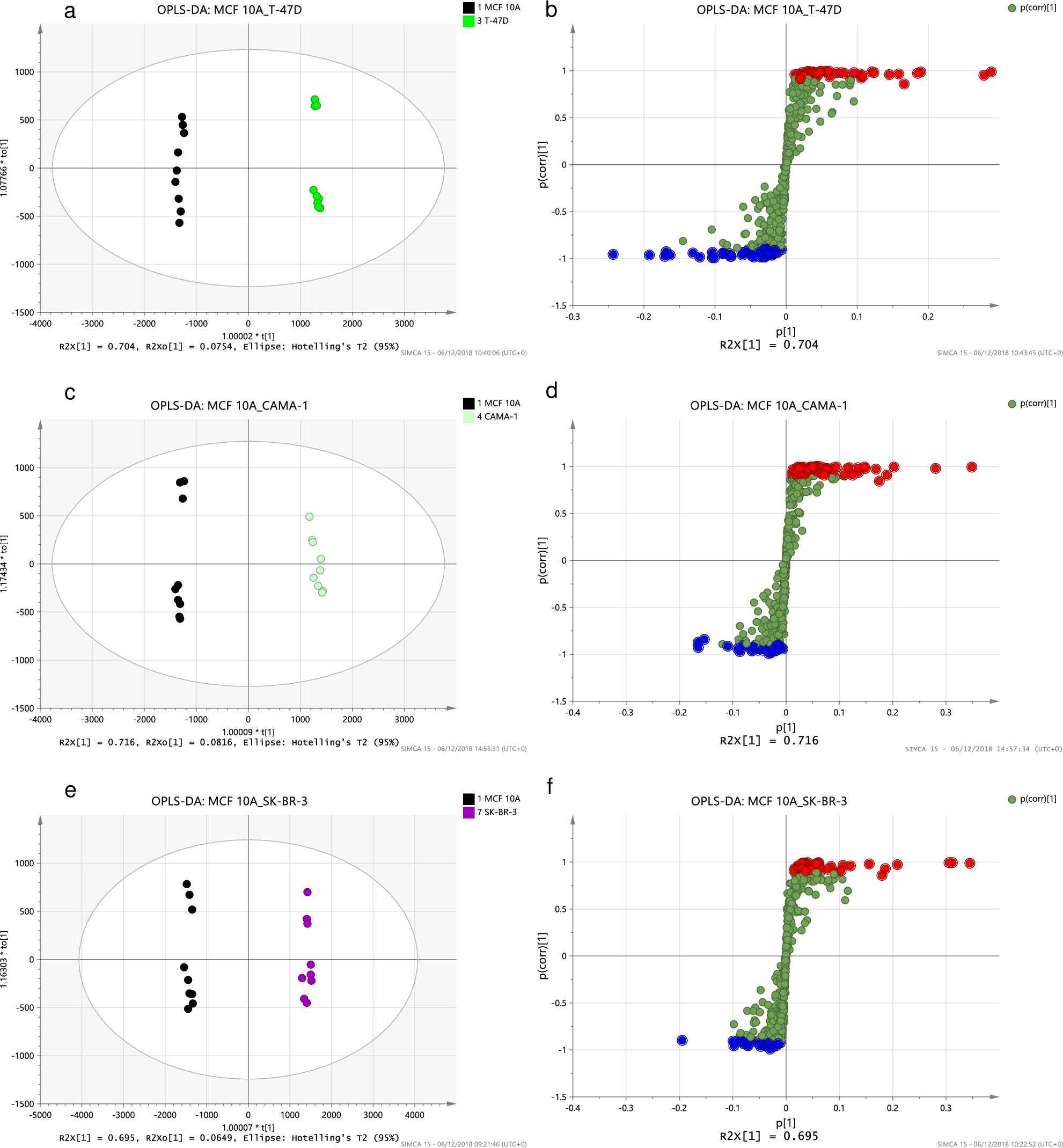

Supplement: S2 Fig — a) OPLS-DA score scatter plot of T-47D cell line compared to reference cell line the reference cell line MCF10A. b) Corresponding S-plot comparing T-47D to the reference cell line MCF10A. c) OPLS-DA score scatter plot of CAMA-1 cell line compared to reference cell line MCF10A. The black spheres represent MCF10A and green represents CAMA-1. d) Corresponding S-plot comparing CAMA-1 to the reference cell line MCF10A. e) OPLS-DA score scatter plot of SK-BR-3 cell line compared to reference cell line MCF10A. The black spheres represent MCF10A and green represents SK-BR-3. f) Corresponding S-plot comparing SK-BR-3 to the reference cell line MCF10A. Scores scatter plots highlight the between class variance in the predictive component on the x-axis (R2Xo [1]) and the within class variation in the orthogonal component on the y-axis (to[1]). In OPLS-DA each green sphere represents an ion feature. The confidence of the ion feature as a discriminant of variance increases with increasing numerical values on the y-axis (-1 or 1) and the size of the contribution increases with increasing numerical values on the x-axis. Ion features selected from S-plots for further identification and processing (cut-off values shown with red dashed lines) are highlighted in red for ion features up-regulated in BC cell line and blue for down-regulated in BC cell line compared to reference. Abundance of 439 ion features normalised to all compounds (CV% ≤ 30%, m/z > 350Da). (TIF) [file pone.0231289.s004.tif]

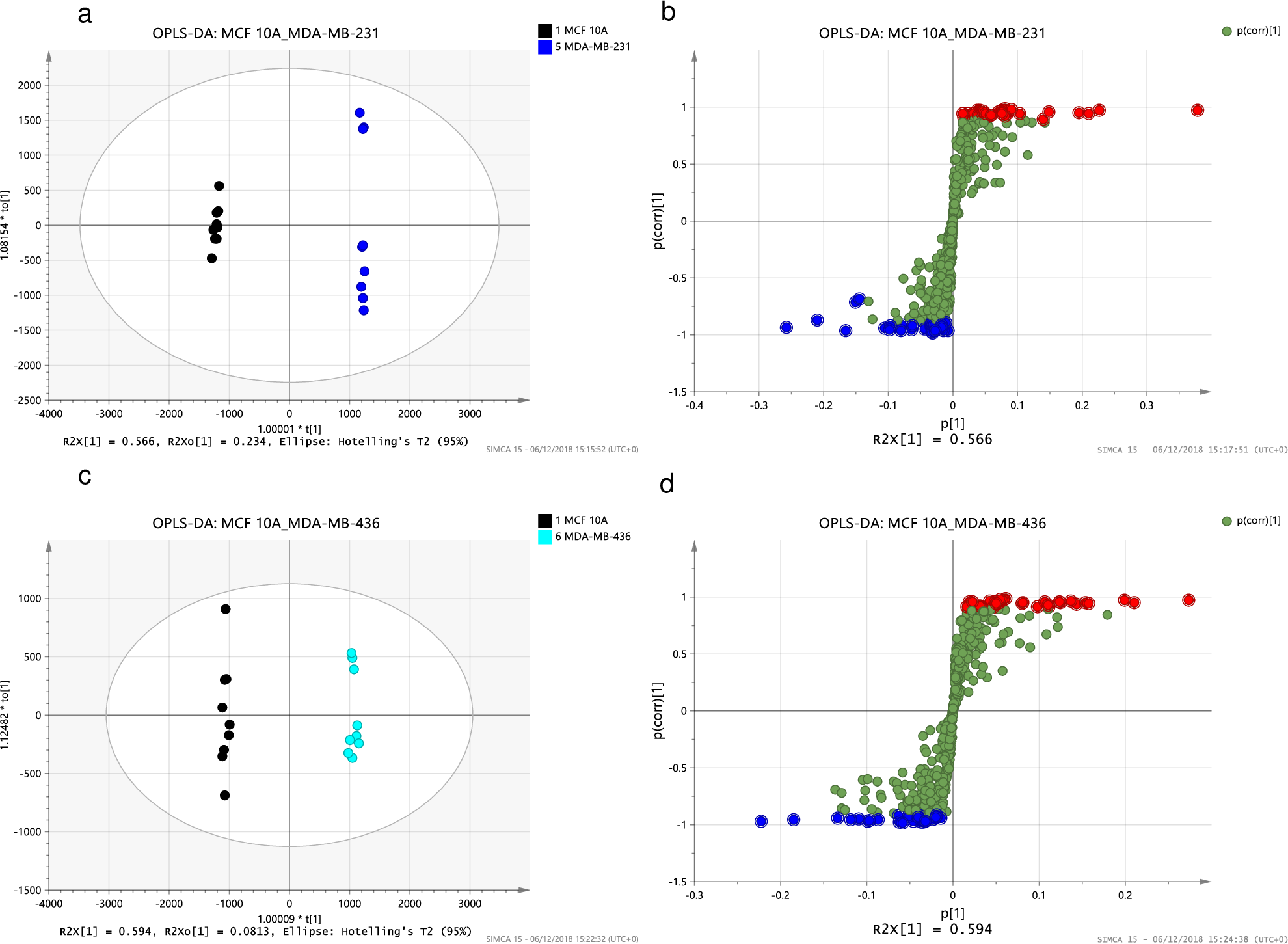

Supplement: S3 Fig — a) OPLS-DA score scatter plot of MDA-MB-231 cell line compared to reference cell line MCF10A. The black spheres represent MCF10A and green represents MDA-MB-231. b) Corresponding S-plot comparing MDA-MB-231 to the reference cell line MCF10A. c) OPLS-DA score scatter plot of MDA-MB-436 cell line compared to reference cell line MCF10A. The black spheres represent MCF10A and green represents MDA-MB-436. d) Corresponding S-plot comparing MDA-MB-436 to the reference cell line MCF10A. Scores scatter plots highlight the between class variance in the predictive component on the x-axis (R2Xo [1]) and the within class variation in the orthogonal component on the y-axis (to[1]). In OPLS-DA each green sphere represents an ion feature. The confidence of the ion feature as a discriminant of variance increases with increasing numerical values on the y-axis (-1 or 1) and the size of the contribution increases with increasing numerical values on the x-axis. Ion features selected from S-plots for further identification and processing (cut-off values shown with red dashed lines) are highlighted in red for ion features up-regulated in BC cell line and blue for down-regulated in BC cell line compared to reference. Abundance of 439 ion features normalised to all compounds (CV% ≤ 30%, m/z > 350Da). (TIF) [file pone.0231289.s005.tif]

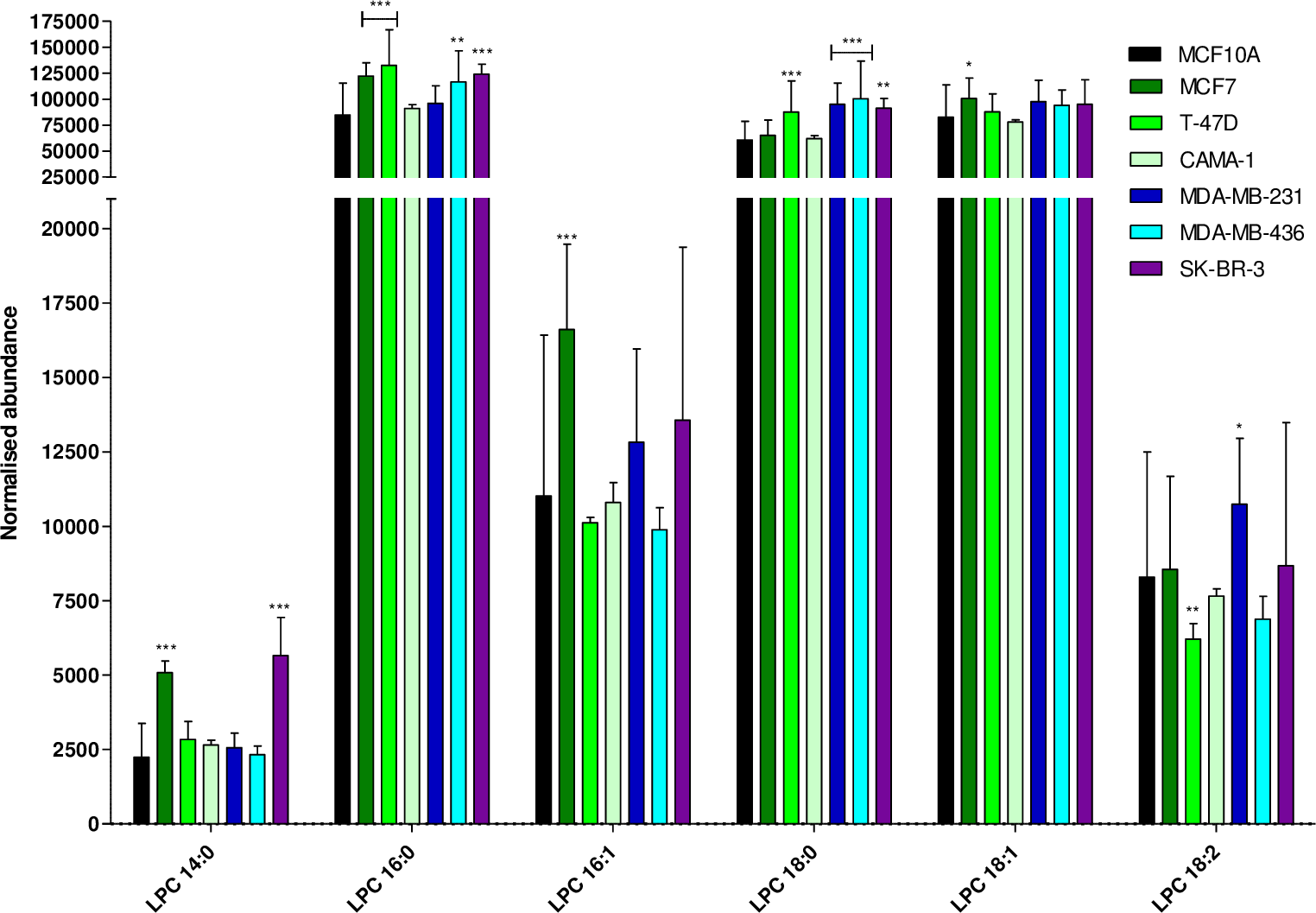

Supplement: S4 Fig — Bars represent mean abundance of three biological replicates, error bars represent SD. Normalised abundance is shown on the y-axis. Statistically significant up- or down-regulation in normalised abundance compared to MCF10A is indicated by * (p< 0.05), ** (p<0.01), *** (p<0.001), insignificant changes are unmarked. (TIF) [file pone.0231289.s006.tif]
